# The Protective Effect of Apamin on LPS/Fat-Induced Atherosclerotic Mice

**DOI:** 10.1155/2012/305454

**Published:** 2012-05-08

**Authors:** Soo-Jung Kim, Ji-Hyun Park, Kyung-Hyun Kim, Woo-Ram Lee, Sok Cheon Pak, Sang-Mi Han, Kwan-Kyu Park

**Affiliations:** ^1^Department of Pathology, School of Medicine, Catholic University of Daegu, 3056-6 Daemyung 4-Dong, Nam-Gu, Daegu 705-718, Republic of Korea; ^2^School of Biomedical Sciences, Charles Sturt University, Bathurst, NSW 2795, Australia; ^3^Department of Agricultural Biology, National Academy of Agricultural Science, Suwon 441-100, Republic of Korea

## Abstract

Apamin, a peptide component of bee venom (BV), has anti-inflammatory properties. However, the molecular mechanisms by which apamin prevents atherosclerosis are not fully understood. We examined the effect of apamin on atherosclerotic mice. Atherosclerotic mice received intraperitoneal (ip) injections of lipopolysaccharide (LPS, 2 mg/kg) to induce atherosclerotic change and were fed an atherogenic diet for 12 weeks. Apamin (0.05 mg/kg) was administered by ip injection. LPS-induced THP-1-derived macrophage inflammation treated with apamin reduced expression of tumor necrosis factor (TNF)-**α**, vascular cell adhesion molecule (VCAM)-1, and intracellular cell adhesion molecule (ICAM)-1, as well as the nuclear factor kappa B (NF-**κ**B) signaling pathway. Apamin decreased the formation of atherosclerotic lesions as assessed by hematoxylin and elastic staining. Treatment with apamin reduced lipids, Ca^2+^ levels, and TNF-**α** in the serum from atherosclerotic mice. Further, apamin significantly attenuated expression of VCAM-1, ICAM-1, TGF-**β**1, and fibronectin in the descending aorta from atherosclerotic mice. These results indicate that apamin plays an important role in monocyte/macrophage inflammatory processing and may be of potential value for preventing atherosclerosis.

## 1. Introduction

Atherosclerosis is a progressive disease characterized by formation of a plaque, consisting mainly of cholesterol, other lipids, and debris from cellular death, in the inner lining of arteries [1]. It is increasingly regarded as a chronic inflammatory disease of the vessel wall [1]. Many macrophages can be observed in atherosclerotic lesions, and early lesions of atherosclerosis are characterized by the infiltration of monocytes/macrophages, proliferation of medial smooth muscle cells, and the presence of macrophage foam cells [2]. Therefore, the number of macrophages in lesions is an important measure of an atherosclerotic plaque.

Macrophages are multipotent inflammatory cells with the capacity to synthesize and secrete proinflammatory cytokines, such as tumor necrosis factor (TNF)-*α*, interleukin (IL)-1*β*, IL-8, and IL-6 [3]. These cytokines play a central role during development of atherosclerosis. Proinflammatory cytokines are regulated at the transcriptional levels by several transcription factors, including activator protein (AP)-1 and nuclear factor-kappa B (NF-*κ*B) [4, 5]. NF-*κ*B is a transcription factor that plays a key role in the regulation of host immune and inflammatory responses by increasing the expression of gene encoding cytokines, chemokines, growth factors, cell adhesion molecules, and several acute phase proteins [6]. NF-*κ*B affects different steps in the atherosclerotic process, including initiation of atherosclerosis, foam cell formation, proliferation of smooth muscle cells, and fibrous cap formation [7]. Proinflammatory cytokines and adhesion molecules, such as vascular cell adhesion molecule (VCAM)-1 and intercellular cell adhesion molecule (ICAM)-1, induce atherosclerosis by NF-*κ*B activation [8, 9]. Cytoplasmic dissociation of NF-*κ*B from inhibitor of *κ*B (I*κ*B) is regulated by activation of I*κ*B inhibitor complex (IKK). Activated IKK phosphorylates I*κ*B*α*, which frees NF-*κ*B dimers to translocate to the nucleus. NF-*κ*B then interacts with *κ*B elements in the promoter region of several inflammatory genes to activate their transcription [10]. Potent inhibitors of IKKs that prevent NF-*κ*B activity through blockage of I*κ*B release can be useful for treatment of inflammatory diseases [11, 12]. Therefore, inhibiting transcription factors related to the activation of inflammation is a good biological target for anti-inflammatory activity.

Apamin is an integral part of bee venom (BV), comprising about 2-3% of its dry weight [13]. BV from the honeybee (*Apis mellifera*) has been traditionally used in China, Korea, and Japan for arthritis, bursitis, back pain, rheumatism, skin disease, and other chronic conditions. BV contains a variety of peptides including melittin, apamin, adolapin, and master cell degranulating peptide. It also contains enzymes, biological amines, and nonpeptide components [14]. It would be interesting to show that apamin in BV is responsible for altering transcription expression of proinflammatory cytokines, although the mechanisms behind that regulation remain unclear. Apamin has long been known as a highly selective blocker of Ca^2+^-activated K^+^ channels [15]. Several studies have confirmed that some calcium channel blockers can decrease areas of atherosclerotic lesions, production of oxidative stress, and expression of inflammatory cytokines without conspicuously effecting blood lipid levels [16]. However, the molecular mechanisms of the anti-atherosclerotic effects of apamin have not been elucidated. To gain a better insight into these mechanisms, the aim of this study is to evaluate the anti-atherosclerotic mechanisms of apamin in THP-1-derived macrophages and to investigate the anti-atherosclerotic effects of apamin in mouse models of atherosclerosis.

## 2. Materials and Methods

### 2.1. Cell Culture

The human monocytic cell line THP-1 was obtained from the American Type Culture Collection and cultured in with RPMI-1640 medium supplemented with 10% fetal bovine serum and 1% antibiotics. Cells were cultured in humidified incubator at 37°C in a 5% CO_2_ atmosphere. THP-1 cells (1 × 10^6^ cells/mL) were differentiated into macrophages with the use of 50 nM phorbol-12-myristate-13-acetate for 48 h. For macrophage inflammation, cells incubated in serum-free culture medium prior to treatment with apamin (0.5, 1, 2 *μ*g/mL, Sigma, MO, USA) for 6 h. After this incubation with apamin, THP-1-derived macrophages were activated with LPS (1 *μ*g/mL, Sigma, MO, USA) for 24 h.

### 2.2. Enzyme-Linked Immunosorbent Assay (ELISA)

Concentrations of TNF-*α* in culture supernatant and serum were measured with a solid-phase sandwich ELISA using a quantikine human or mouse TNF-*α* kit (R&D Systems, MN, USA). The absorbance was measured at 450 nm in an ELISA reader (BMG labtechnologies, Mornington, Germany).

### 2.3. Western Blot Analysis

Cells or tissues were homogenized in a lysis buffer (50 mM Tris pH 8.0, 150 mM NaCl, 5 mM EDTA, 0.5% NP-40, 100 mM PMSF, 1 M DTT, 10 mg/mL leupeptin, and aprotinin; all from Sigma, MO, USA). For cytosolic fractions, cells were suspended in extraction buffer (10 mM HEPES pH 8.0, 1.5 mM MgCl_2_, 10 mM KCl, 0.5 mM DTT, 300 mM sucrose, 0.1% NP-40, and 0.5 mM PMSF) for 15 min on ice and were centrifuged 6000 × g for 15 min. The supernatant from this step is the cytosolic fraction, and the pellet is the nuclear fraction. The nuclear fractions were collected by different extraction buffer (20 mM HEPES pH 8.0, 20% glycerol, 100 mM KCl, 100 mM NaCl, 0.2 mM EDTA, 0.5 mM PMSF, and 0.5 mM DTT) for 15 min on ice. The nuclear fractions were centrifuged 12000 × g for 10 min at 4°C to remove insoluble protein. Then, protein concentration was determined using the Bradford assay. Total protein was separated on 10% to 12% SDS-polyacrylamide gels and transferred to polyvinylidene fluoride membrane (Millipore, MA, USA). Membranes were blocked in 5% skim milk for 1 h at room temperature. Protein samples were incubated with primary antibodies for 3 h. Primary antibodies used in this study were the following: anti-VCAM-1, anti-ICAM-1, and anti-TGF-*β*1 were obtained from R&D Systems (MN, USA); anti-IKK, anti-phospho-IKK, anti-I*κ*B*α*, anti-phospho-I*κ*B*α*, anti-NF-*κ*B p65 and anti-phospho-NF-*κ*B p65 were purchased from Cell Signaling Technology (MA, USA); anti-fibronectin and anti-F4/80 were obtained from Abcam (MA, USA), anti-glyceraldehyde-3-phosphate dehydrogenase (GAPDH) and histone H2B were purchased from Santa Cruz (CA, USA). The membranes probed with a horseradish peroxidase (HRPO)-conjugated secondary antibodied were used for detection. Target proteins were detected using an enhanced chemiluminescence detection system (Amersham, NJ, USA) and film.

### 2.4. Electrophoretic Mobility Shift Assay (EMSA)

A DIG Gel shift kit (Roche, Mannheim, Germany) was used for EMSA assays. The NF-*κ*B oligonucleotide probe (5′-CTT GAA GGG ATT TCC CTG GCT TGA AGG GAT TTC CCT GG-3′; only sense strands are shown) was end-labeled with DIG-ddUTP. For binding reaction, 9 *μ*g nuclear extract protein was mixed with binding buffer (0.5 *μ*g poly dI-dC, 0.1 *μ*g poly L-lysine, and 0.8 *μ*g labeled oligonucleotide, final volume of 20 *μ*L) and was incubated at 37°C for 30 min. The nuclear protein-DNA complex was separated by 6% non-denaturing polyacrylamide gel in TBE buffer (22.5 mM Tris, 22.5 mM boric acid, 0.5 mM EDTA, and pH 8.3) at 80 V for 1.5–2 h at 4°C. Samples were transferred to Hybond-XL membranes (Amersham Biosciences, Amersham, UK) for 30 min and crosslinked for 10 min under ultraviolet light. Membrane was incubated with anti-digoxigenin-AP Fab fragments (1 : 10,000) for 30 min, and the nuclear protein-DNA bands were developed with detection solution including 100 mM Tris-HCl, 100 mM NaCl, pH 9.5, and 100 *μ*g/mL disodium 3-[4-metoxyspiro {1,2-dioxetane-3,2′(5′-chloro)tricyclo(3.3.1.1^3,7^)decan}-4-yl] phenyl phosphate. 

### 2.5. NF-*κ*B Promoter Activity Assay

Reporter gene activity was evaluated by cell-based analysis methods for assaying NF-*κ*B activity. NF-*κ*B promoter-luciferase construct was transiently transfected by using transfection reagent lipofectamine 2000 (Invitrogen, CA, USA). After harvesting, cells were lysed in reporter lysis buffer (Promega, WI, USA), cell extract (50 *μ*g/20 *μ*L) was mixed with 100 *μ*L of luciferase assay reagent, and the emitted light intensity was measured using luminometer FLUOstar OPIMA (BMG Labtech, Germany). The luciferase activity was represented as the fold induction compared with normal control cells.

### 2.6. Experimental Animals

Male C57BL/6 mice (8 weeks old, 20–25 g) were obtained from Samtako (Osan, Republic of Korea) and were allowed one week for stabilization. Mice were kept in a room maintained at 21–25°C under 12 h dark/light cycles. The animal experiments were performed in accordance with the NIH guidelines for the care and use of laboratory animals. Mice were randomly subdivided into four groups (*n* = 10/group) and were maintained under various conditions for 12 weeks. The normal control (NC) group was fed with chow diet (Samyang Feed, Daejeon, Republic of Korea). The apamin (Apa) group was fed with chow diet and ip injected with 0.05 mg/kg apamin (Sigma, MO, USA) twice a week. The LPS/fat group (atherosclerotic mice) was fed with an atherogenic diet (1.25% cholesterol, 15% fat, and 0.5% cholic acid) and ip injected with 2 mg/kg LPS (Sigma, MO, USA) three times a week. The LPS/fat+Apa group was atherosclerotic mice treated with 0.05 mg/kg apamin twice a week.

### 2.7. Biochemical Analysis

Blood was collected from inferior vena cava and immediately centrifuged at 8000 × g for 10 min at 4°C to separate serum. Serum total cholesterol (TC) and triglycerides (TG) were measured using a commercial kit (Asan, Hwaseong, Republic of Korea). Serum Ca^2+^ accumulation was measured using a commercial kit (BioAssay Systems, CA, USA). The concentration of Ca^2+^ accumulation was determined with reference to a standard curve constructed with each assay, and mean plus standard deviation was calculated.

### 2.8. Reverse-Transcription Polymerase Chain Reaction (RT-PCR)

Total RNA was isolated from the aorta with TRIzol Reagent (Gibco, NY, USA) according to manufacturer's recommendations. RNA (0.5 *μ*g) was reverse-transcribed using M-MLV reverse transcriptase (Promega, WI, USA). Single-stranded cDNA was amplified by PCR with primers (Bioneer, Daejeon, Republic of Korea) specific to mouse VCAM-1, ICAM-1, TGF-*β*1, fibronectin, and GAPDH used as a positive control. Primer sequences are the following: ICAM-1 sense: 5′-AGC ACC TCC CCA CCT ACT TT-3′; ICAM-1 antisense: 5′-AGC TTG CAC GAC CCT TCT AA-3′; VCAM-1 sense: 5′-TAC CAG CTC CCA AAA TCC TG-3′; VCAM-1 antisense: 5′-TCT GCT AAT TCC AGC CTC GT-3′; TGF-*β*1 sense: 5′-CCT GCT GCT TTC TCC CTC AAC C-3′; TGF-*β*1 antisense: 5′-CTG GCA CTG CTT CCC GAA TGT C-3′; fibronectin sense: 5′-TGT GAC AAC TGC CGT AGA CC-3′; fibronectin antisense: 5′-GAC CAA CTG TCA CCA TTG AGG-3′; GAPDH sense: 5′-GTG GAC ATT GTT GCC ATC AAC G-3′; GAPDH antisense: 5′-GAG GGA GTT GTC ATA TTT CTC G-3′. PCR products were visualized by 2% agarose gel. The band intensity was quantified by using Image Analysis (Las 3000, Fuji, Japan).

### 2.9. Histological Analysis

All aorta specimens were fixed overnight in 10% formalin solution, dehydrated, and embedded in paraffin. Thin sections were mounted on glass slides, dewaxed, rehydrated in distilled water, and stained with hematoxylin and eosin (H&E) and Verhoeff' elastic tissue staining solution (alcoholic hematoxylin, 10% ferric chloride and iodine solution). For immunohistochemistry, sections were incubated with anti-ICAM-1 (R&D Systems, MA, USA) and anti-F4/80 (Abcam, MA, USA) for 1 h at 37°C. Signals were visualized using an Envision system (DAKO, CA, USA) for 30 min at 37°C. DAB (3,3′-diaminobenzidine tetrahydrochloride) was used as the coloring reagent, and hematoxylin was used as counterstain. For immunofluorescence staining, aorta sections were incubated with anti-F4/80 (Abcam, MA, USA) and anti-mouse biotinylated secondary antibodies conjugated with FITC (Jackson ImmunoResearch Laboratories, PA, USA). Slides were mounted using VECTASHIELD Mounting Medium (VECTOR Laboratories, CA, USA). Specimens were examined and photographed using a fluorescence microscope. Sections were counterstained with Hoechst 33342 (Immunochemistry, MN, USA).

### 2.10. Statistical Analysis

Data were collected from three independent experiments and analyzed with SPSS 12.0 software (SPSS Inc., IL, USA). Results were expressed as mean ± SD, and *P* value < 0.05 was considered as statistical significance.

## 3. Results

### 3.1. Apamin Inhibits Expression of Proinflammatory Cytokine and Adhesion Molecules

**Figure 1:**
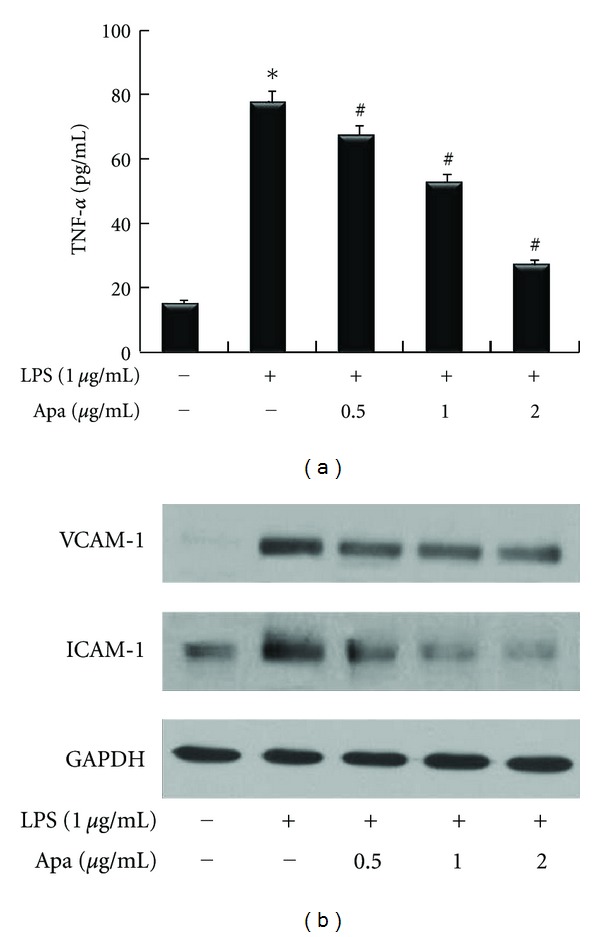
The effect of apamin on expression levels of proinflammatory cytokine and adhesion molecules in LPS-treated THP-1-derived macrophages. (a) Expression levels of TNF-*α* in culture supernatant, as determined by ELISA, significantly inhibited by apamin in a dose-dependent manner. (b) Expression levels of VCAM-1 and ICAM-1 were determined by western blot. They were decreased by apamin in a dose-dependent manner. As a loading control, GAPDH was used to confirm equal sample loading. *P < 0.05 compared to control cells, ^#^ *P* < 0.05 compared to control cells treated with LPS alone.

To investigate the effect of apamin on inflammatory response, this study assessed the effect of apamin on LPS-induced cytokine secretion in THP-1-derived macrophages (Figure 1(a)). Expression levels of proinflammatory cytokine were validated by an ELISA kit. THP-1-derived macrophages expressed TNF-*α* after exposure to LPS. Upregulation of TNF-*α* in LPS-treated THP-1-derived macrophages was suppressed by apamin in a concentration-dependent manner. Expression levels of adhesion molecules, including VCAM-1 and ICAM-1, were determined by western blot (Figure 1(b)). Protein levels of VCAM-1 and ICAM-1 were higher in LPS-treated THP-1-derived macrophages than in normal control cells. Treatment with apamin resulted predominantly in the dose-dependent downregulation of VCAM-1 and ICAM-1 expression levels in response to LPS. These results indicate that apamin efficiently discourages the activity of proinflammatory cytokine and adhesion molecules in THP-1-derived macrophages.

### 3.2. Apamin Inhibits NF-*κ*B Activation and Nuclear Translocation of NF-*κ*B

**Figure 2:**
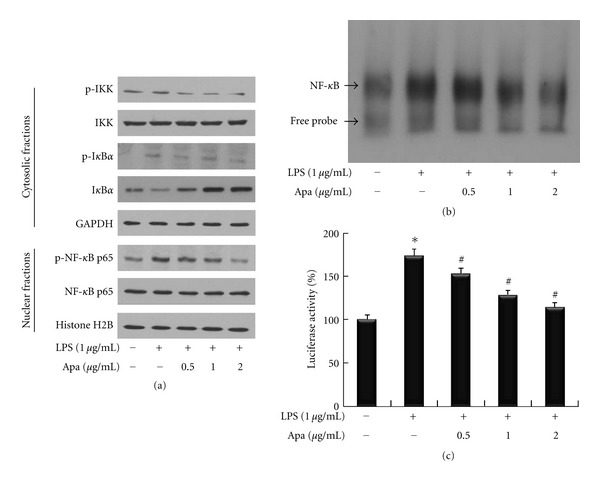
The effect of apamin on NF-*κ*B signaling pathway in LPS-treated THP-1-derived macrophages. (a) Expression levels of IKK and I*κ*B*α* in the cytosolic fraction and NF-*κ*B in the nuclear fraction were determined by western blot. GAPDH and histone H2B were used as the internal controls for cytosolic and nuclear fraction loading control, respectively. (b) Nuclear NF-*κ*B activity was examined by EMSA. The arrow indicates the specific NF-*κ*B band. (c) Luciferase activity was measured with a luminometer. *P < 0.05 compared to control cells, ^#^ *P* < 0.05 compared to control cells treated with LPS alone.

To determine the involvement of the IKK, I*κ*B, and NF-*κ*B signaling pathways in the anti-inflammatory property of apamin, activation of these three proteins was examined by western blots of their dually phosphorylated forms (Figure 2(a)). Upon LPS treatment, the total IKK protein did not change from the cytosolic fractions, whereas the phosphorylated IKK protein was particularly increased when compared to the normal control. Addition of apamin inhibited the LPS-induced phosphorylated IKK expression level. Expression of I*κ*B phosphorylation tended to increase when treated with LPS. Addition of apamin reduced LPS-induced I*κ*B phosphorylation. In the nuclear fraction, phosphorylated NF-*κ*B was also inhibited by apamin in a dose-dependent manner. To determine whether the inhibitory effect of apamin on LPS-induced NF-*κ*B activation was due to inhibition of I*κ*B phosphorylation and NF-*κ*B translocation, nuclear NF-*κ*B p65 subunit levels were measured following treatment with LPS in the presence and absence of apamin. While LPS treatment increased nuclear NF-*κ*B p65, apamin cotreatment suppressed this translocation. These results supported the explanation that apamin inhibits NF-*κ*B activation by suppression of I*κ*B phosphorylation at the transcription level in LPS-treated THP-1-derived macrophages. After demonstrating that apamin decreased NF-*κ*B protein expression as measured by western blot, the next experiment attempted to further examine the effect of apamin on transcriptional activity of NF-*κ*B by EMSA. The DNA-binding activity of NF-*κ*B nuclear protein was markedly higher in LPS-treated THP-1-derived macrophages. LPS-induced NF-*κ*B nuclear protein-DNA binding activity was noticeably inhibited by apamin in a dose-dependent manner (Figure 2(b)). To investigate the transcriptional activity of NF-*κ*B, expression of reporter genes in cells transfected with plasmid NF-*κ*B was analyzed. As shown in Figure 2(c), treatment of THP-1-derived macrophages with LPS resulted in increased NF-*κ*B activity that was suppressed by apamin in a dose-dependent manner. The results were consistent view that apamin inhibits the expression of NF-*κ*B, probably at the transcriptional level.

### 3.3. Apamin Reduces Lipid Levels, Ca^2+^Accumulation, and TNF-*α* Expression of Serum

**Figure 3:**
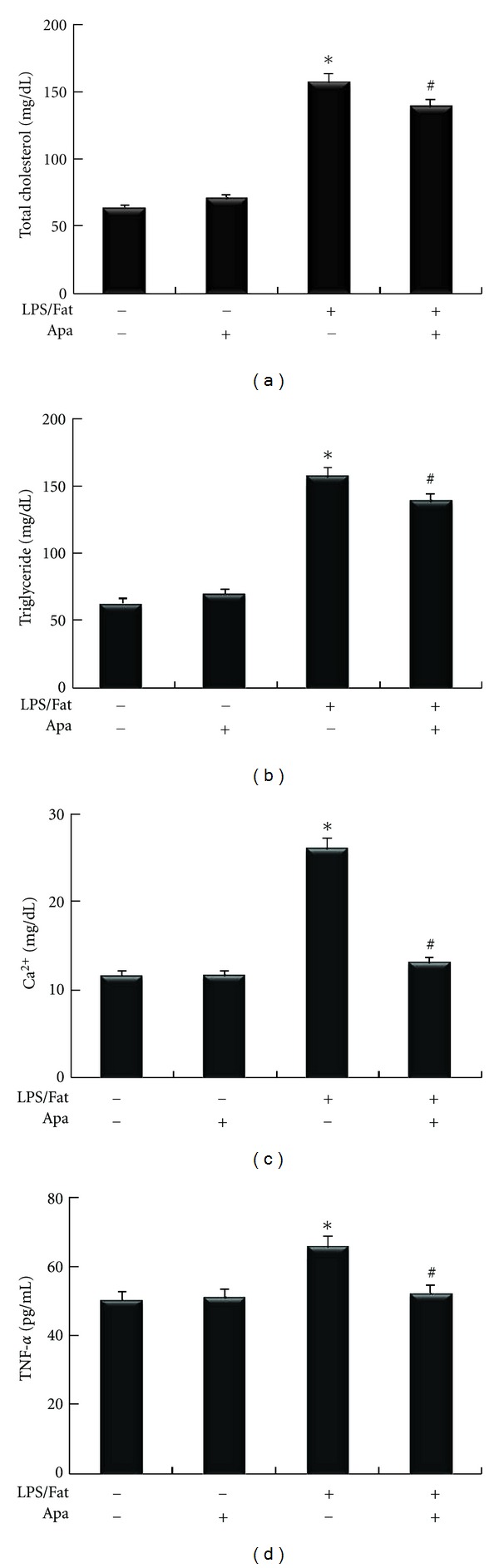
The effect of apamin on biochemical parameters in atherosclerotic mice. Total cholesterol (a), triglycerides (b), and Ca^2+^ accumulation (c) were measured using a commercial kit. Expression level of TNF-*α* (d) was determined by ELISA. *P < 0.05 compared to normal control, ^#^ *P* < 0.05 compared to atherosclerotic mice.

The effects of apamin on serum TC and TG levels were determined. After 12 weeks of feeding a high-fat diet and LPS treatment, mice developed severe atherosclerosis with significant elevation of serum TC and TG levels, compared to the NC and Apa groups. TC and TG levels were significantly decreased by apamin (Figures 3(a) and 3(b)). To confirm that apamin affected Ca^2+^accumulation in atherosclerotic mice, Ca^2+^ accumulation of serum was measured. As shown in Figure 3(c), Ca^2+^ accumulation was markedly enhanced in the LPS/fat group compared to the NC and Apa groups. Ca^2+^ accumulation was predominantly reduced in serum from apamin-treated atherosclerotic mice compared to the LPS/fat group. The expression level of TNF-*α* was measured by an ELISA kit. The NC and Apa groups did not display a significant difference in expression level of TNF-*α* (Figure 3(d)). Expression level of TNF-*α* was elevated in the LPS/Fat group. In atherosclerotic mice treated with apamin, expression level of TNF-*α* was reduced compared to the LPS/Fat group.

### 3.4. Apamin Attenuates Formation of Atherosclerotic Lesions

**Figure 4:**
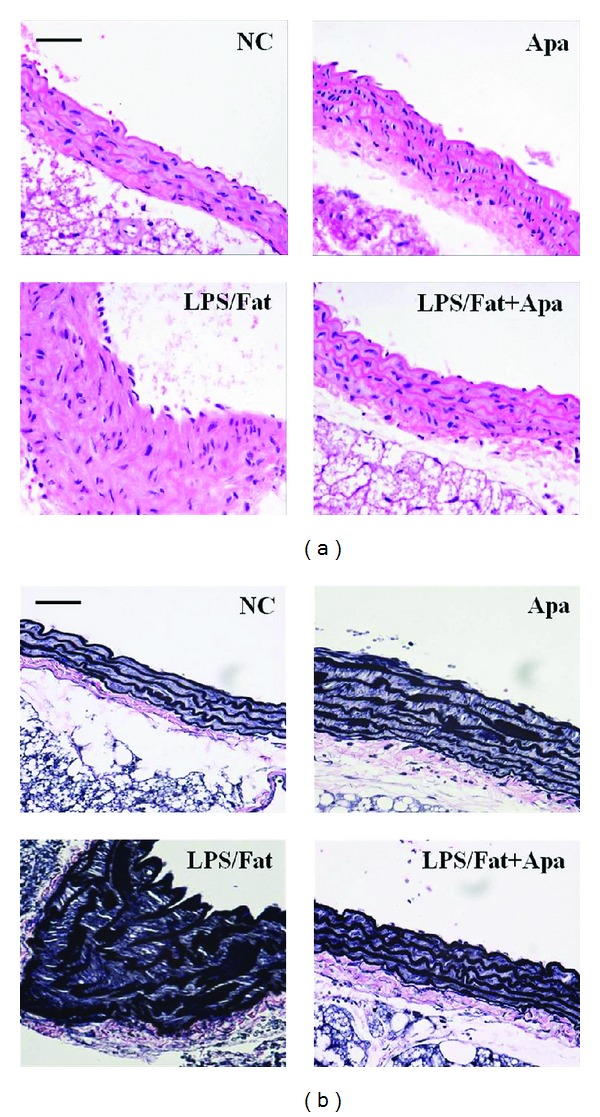
Histological cross-sections of descending aorta in atherosclerotic mice. (a) H&E staining and (b) elastic staining showed that atherosclerotic changes were attenuated in the LPS/Fat+Apa group by apamin. NC, normal control; Apa, normal control treated with apamin; LPS/Fat, LPS injection and high fat dieted mice (atherosclerotic mice); LPS/Fat+Apa, atherosclerotic mice treated with apamin. Scale bars, 50 *μ*m.

Pathological evaluations of aortic lesions were carried out. Consecutive cross-sections of cuffed descending aortas stained with H&E revealed induction of atherosclerotic lesion formation. The LPS/fat group showed a larger number of atherosclerotic lesions in the aorta compared to the NC and Apa groups (Figure 4(a)). In the LPS/fat group, nearly all animals developed fatty streaks in the aortic arch, with accumulation of lipids localized mainly in areas subjacent to the endothelium. Compared to the LPS/fat group, treatment with apamin changed the size of atherosclerotic lesions in aortas suggesting that apamin exerted apparent protective atherogenic actions. These inhibitory effects of apamin on atherosclerotic mice were markedly displayed among the four groups when entire aortas were stained by elastic stain (Figure 4(b)). In atherosclerotic mice treated with apamin, atherosclerotic lesions were significantly decreased compared to the LPS/fat group. This result was consistent with H&E stain analysis.

### 3.5. Apamin Attenuates Expression Levels of Adhesion Molecules and Fibrotic Factors

**Figure 5:**
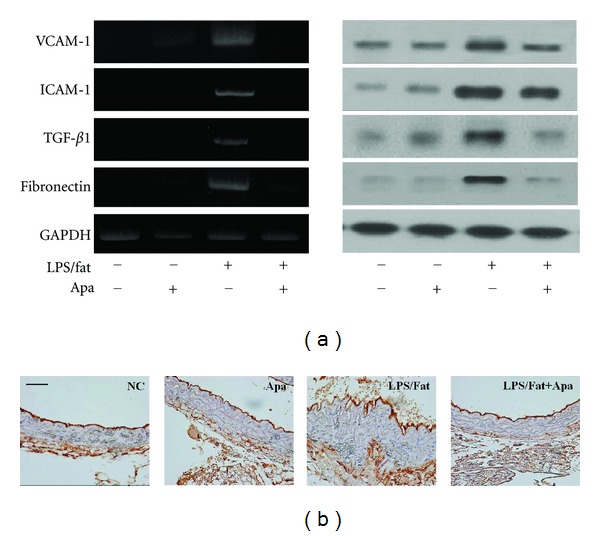
The effect of apamin on expression levels of VCAM-1, ICAM-1, TGF-*β*1, and fibronectin in atherosclerotic mice. (a) Western blot (left panel) and RT-PCR (right panel) suppressed expression levels of those protein by apamin. As a loading control, GAPDH was used to confirm equal sample loading. (b) Immunohistochemistry of ICAM-1 predominantly attenuated in the LPS/fat+Apa group. NC, normal control; Apa, normal control treated with apamin; LPS/fat, LPS injection and high-fat-dieted mice (atherosclerotic mice); LPS/fat+Apa, atherosclerotic mice treated with apamin. Scale bars, 50 *μ*m.

Protein and mRNA levels of adhesion molecules (VCAM-1 and ICAM-1) and fibrotic factors (TGF-*β*1 and fibronectin) after apamin treatment in atherosclerotic mice were detected by western blot and RT-PCR, respectively. As shown in Figure 5(a), aortas from the NC, and Apa groups showed little expression of VCAM-1 and ICAM-1. However, a more significant upregulation of these expression levels was observed in the LPS/Fat group, while treatment with apamin led to evident downregulation of VCAM-1 and ICAM-1 expression levels. Similarly, expression levels of TGF-*β*1 and fibronectin were decreased in the aorta of the LPS/fat+Apa group compared to LPS/fat group. Moreover, expression levels of ICAM-1 in atherosclerotic lesions were determined by immunohistochemical staining. As shown in Figure 5(b), ICAM-1 expression levels were barely detected in aortic sections from the NC and Apa groups. Suppressed expression levels in surface area lesions revealed that apamin inhibited ICAM-1 expression levels in atherosclerotic lesions, which agreed with the results from current *in vitro* experiment. These results indicated that apamin suppresses the expression of VCAM-1, ICAM-1, TGF-*β*1, and fibronectin in atherosclerotic mice.

### 3.6. Apamin Reduces Infiltration of Macrophages

**Figure 6:**
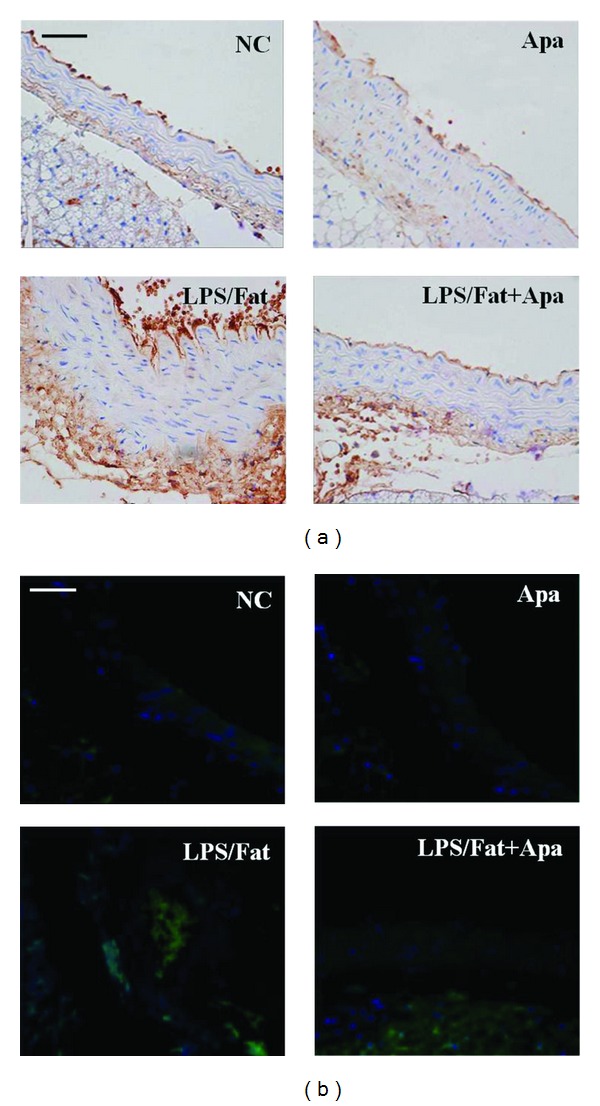
The effect of apamin on macrophage infiltrations in atherosclerotic mice. (a) Immunohistochemistry and (b) immunofluorescence examinations showed that apamin markedly inhibited macrophages infiltration in atherosclerotic mice. Micrographs display macrophages (green), nuclei (blue), and merged images. NC, normal control; Apa, normal control treated with apamin; LPS/Fat, LPS injection and high-fat-dieted mice (atherosclerotic mice); LPS/Fat+Apa, atherosclerotic mice treated with apamin. Scale bars, 50 *μ*m.

To investigate whether apamin could influence atherosclerotic lesions, aortas of experimental mice were collected for immunohistochemistry. Greater macrophage infiltrations in atherosclerotic mice were demonstrated using F4/80, a specific marker of macrophages (Figure 6(a)). Atherosclerotic lesions in atherosclerotic mice treated with apamin showed decreased infiltration of macrophages into the arterial wall. F4/80 positive areas in the mice treated with apamin were significantly reduced to the total cross-sectional area of aortas compared to the LPS/fat group. Similarly, macrophages infiltration of atherosclerotic lesions was markedly attenuated by apamin, as determined by immunofluorescence staining (Figure 6(b)). Taken together, these results indicate that apamin treatment substantially attenuates atherosclerotic lesions.

## 4. Discussion

Atherosclerosis is increasingly recognized as a chronic inflammatory disease. It is a multifactorial and progressive disease in which inflammatory reaction and inflammation-related mediators play pivotal roles at all stages [17]. Various research studies of the anti-inflammatory effect of BV have recently been conducted [18–20]. Studies of the general pharmacological profiles of BV and its components have also been conducted [13, 21]. Apamin comprises 2-3% of the dry weight of apamin [22]. BV has been reported as having proinflammatory [23] and anti-inflammatory effects [18]. A number of studies have reported on a variety of mechanisms for the anti-inflammatory effect of BV and its constituents [13, 19, 22]. An anti-inflammatory effect of apamin accompanied by a reduction of seromucoid and haptoglobin levels has been reported [22].

In this study, apamin suppressed LPS-induced THP-1-derived macrophage inflammation via the NF-*κ*B signal pathway. Furthermore, in atherosclerotic mice, apamin attenuated the regulation of various atherosclerotic factors by inhibiting inflammation.

Inflammation is pivotal to atherosclerosis, and monocytes/macrophages are critical participants. Monocytes/macrophages secrete IL-6, IL-1*β*, and TNF-*α*, and the serum concentrations of several markers of inflammation are associated with future cardiovascular risk [24]. This study used an *in vitro* cell-based system using THP-1-derived macrophages with LPS stimulation to test the anti-inflammatory effect of apamin. Apamin treatment significantly decreased TNF-*α* expression level in LPS-induced THP-1-derived macrophages. Adhesion molecules also play important roles in cellular interactions during inflammatory responses. Expression of VCAM-1 and ICAM-1 may influence the organization of cells that promote the production of cytokines in inflammatory cells [3]. Our data proves that LPS stimulation promotes the expression of these adhesion molecules in THP-1-derived macrophages. The expression levels of VCAM-1 and ICAM-1 were predominantly reduced in a dose-dependent manner after treatment with apamin in mice.

A role for activation of the NF-*κ*B in regulation of inflammatory responses was reported [25]. NF-*κ*B activation by TNF-*α* is required for transcription of the gene encoding cell adhesion molecules [26]. Following exposure of macrophages to LPS, NF-*κ*B is activated by phosphorylation and degradation of I*κ*B. The activated NF-*κ*B was then translocated into the nucleus, leading to transcriptional expression of genes associated with inflammatory responses [27]. In the current study, apamin suppressed LPS-inducible I*κ*B phosphorylation and nuclear NF-*κ*B p65 activation in a concentration-dependent manner in THP-1-derived macrophages. These results demonstrate that apamin mediated an anti-inflammatory effect via the NF-*κ*B signaling system. More data is required to determine whether the potential differential effect of apamin occurs upstream of NF-*κ*B and particularly upstream of IKK based on current results that apamin inhibited phosphorylation of IKK in a dose-dependent manner in THP-1-derived macrophages.

Based on these *in vitro* results, the effect of apamin in an animal model of atherosclerosis was investigated. Atherosclerosis can be induced in mice given LPS injection and fed a high-fat diet [20, 28]. Accumulation of cholesterol and lipids resulting in foam cell formation is regarded as a critical process in the development of atherosclerosis. Progressive lipid accumulation leads to increases in expression of proinflammatory cytokines and infiltration of inflammatory cells [29]. Proinflammatory cytokines have been reported to promote efflux of cholesterol [30]. Stimulation of the mechanism involved in decrease of lipids and cholesterol in macrophages may thus be an effective way to prevent atherosclerosis. Our results from treatment with apamin showed that serum TC and TG levels were significantly decreased in the atherosclerotic mice. Studies have demonstrated that modification of lipid can induce Ca^2+^ influx, that Ca^2+^ is closely associated with production of TNF-*α* involved in the inflammatory response, and that inhibition of the influx of Ca^2+^ might attenuate these responses [31, 32]. In this study, apamin significantly reduced Ca^2+^ accumulation. In addition, TNF-*α* level decreased in the serum of atherosclerotic mice following apamin treatment. These data suggest that the anti-atheroscleortic effects of apamin may occur via inhibition of Ca^2+^ accumulation and proinflammatory cytokines.

Expression levels of VCAM-1 and ICAM-1 were largely studied in a mouse model of atherosclerosis [33], and these molecules were upregulated by a high-cholesterol diet in an animal model [34]. Presently, protein and mRNA levels of these adhesion molecule expressions were predominantly decreased by apamin in atherosclerotic mice. Further, atherosclerotic lesion development was accompanied by upregulation of ICAM-1. This is consistent with other studies that reported that a high-fat diet induces both lesion development and expression of adhesion molecules in general [33, 34]. Treatment with apamin attenuated both lesion development and adhesion molecule expressions in atherosclerotic mice.

TGF-*β*1 expression is upregulated in plaque development and appears to be involved in atherosclerotic lesions under a variety of circumstances which is further related to extracellular matrix remodeling [35]. The extracellular matrix protein fibronectin is focally deposited in atherosclerosis where it contributes to inflammatory signaling [36]. Several studies have reported that monocyte binding to fibronectin causes the induction of potent proinflammatory cytokines, together with the ability of fibronectin to promote NF-*κ*B activation [36, 37]. In the present study, protein and mRNA levels of TGF-*β*1 and fibronectin expression were inhibited by apamin in atherosclerotic mice.

Infiltration and activation of monocytes into the arterial walls are critical steps in atherogenesis [38]. Macrophages accumulate cholesterol and lipids, resulting in foam cell formation, which is considered a critical process in development of atherosclerosis [39]. We were able to witness the macrophage infiltration via western blot (data not shown), immunohistochemistry, and immunofluoresence staining. However, macrophage infiltration was significantly reduced by apamin in the aorta from atherosclerotic mice.

## 5. Conclusion

Treatment with apamin in THP-1-derived macrophages suppresses inflammatory responses by a decrease of the NF-*κ*B signal pathway. Furthermore, atherosclerotic mice treated with apamin predominantly attenuate lipids, Ca^2+^ levels, proinflammatory cytokines, adhesion molecules, fibrotic factors, and macrophage infiltration. These mechanisms could be involved in the possible role of apamin in protection against atherosclerosis. Therefore, apamin may be a therapeutically useful agent for use in atherosclerosis prevention.
